# Computational multifocus fluorescence microscopy for three-dimensional visualization of multicellular tumor spheroids

**DOI:** 10.1117/1.JBO.27.6.066501

**Published:** 2022-06-02

**Authors:** Julia R. Alonso, Alejandro Silva, Ariel Fernández, Miguel Arocena

**Affiliations:** a Universidad de la República, Instituto de Física, Facultad de Ingeniería, Montevideo, Uruguay; b Instituto de Investigaciones Biológicas Clemente Estable, Departamento de Genómica, Montevideo, Uruguay; c Universidad de la República, Cátedra de Bioquímica y Biofísica, Facultad de Odontología, Montevideo, Uruguay

**Keywords:** fluorescence microscopy, three-dimensional visualization, stereoscopic pairs, computational optical imaging

## Abstract

**Significance:**

Three-dimensional (3D) visualization of multicellular tumor spheroids (MCTS) in fluorescence microscopy can rapidly provide qualitative morphological information about the architecture of these cellular aggregates, which can recapitulate key aspects of their *in vivo* counterpart.

**Aim:**

The present work is aimed at overcoming the shallow depth-of-field (DoF) limitation in fluorescence microscopy while achieving 3D visualization of thick biological samples under study.

**Approach:**

A custom-built fluorescence microscope with an electrically focus-tunable lens was developed to optically sweep in-depth the structure of MCTS. Acquired multifocus stacks were combined by means of postprocessing algorithms performed in the Fourier domain.

**Results:**

Images with relevant characteristics as extended DoF, stereoscopic pairs as well as reconstructed viewpoints of MCTS were obtained without segmentation of the focused regions or estimation of the depth map. The reconstructed images allowed us to observe the 3D morphology of cell aggregates.

**Conclusions:**

Computational multifocus fluorescence microscopy can provide 3D visualization in MCTS. This tool is a promising development in assessing the morphological structure of different cellular aggregates while preserving a robust yet simple optical setup.

## 1 Introduction

Three-dimensional (3D) culture of cancer cells mimics the *in vivo* microenvironment more closely compared to two-dimensional (2D) monolayer cell culture (e.g., in a petri dish). In this regard, imaging of cell aggregates known as 3D multicellular tumor spheroids (MCTS) is of high relevance.^1^ MCTS recapitulate key parameters of the tumor microenvironment, such as gradients of hypoxia and extracellular pH, which makes them a more realistic model of the early tumor environment than the standard methodology of 2D cell culture.^2^ Since most cellular components are colorless, to observe for example the nuclei in MCTS, cells are usually stained with DNA binding fluorescent probes such as 4’,6-diamidino-2-phenylindole (DAPI) and observed through a fluorescence microscope.^3^ Fluorescent staining of DNA by DAPI then allows to visualize of cell nuclei within 3D MCTS^4^ to provide morphological information about the architecture of the MCTS.^5^

However, limited depth-of-field (DoF) emerges as an optical limitation which makes it impossible for the all-in-focus visualization of the 3D structure of a thick sample in a single image. One way to achieve 3D fluorescence imaging is by means of optical sectioning in confocal,^6^ in structured illumination,^7^ or in light-sheet microscopy^8^ at the cost of rather complex optical setup and calibration. Digital holography has also been proposed for fluorescence microscopy^9^ to retrieve 3D information by incorporating a reference beam into the setup with a consequent extra alignment in the system. Transport of irradiance equation ^10^ is an alternative, noninterferometric technique, where phase distribution needs also to be retrieved from defocused fluorescence images to estimate, after inverse Fresnel propagation, focused images at different planes. Other methods are based on acquiring spatially multiplexed information from the sample. This is the case for light-field microscopy where a microlens array is inserted in front of the microscope’s image sensor to simultaneously capture 2D spatial and 2D angular information,^11^^–^^13^ integral imaging,^14^^,^^15^ plenoptic projection fluorescence tomography,^16^ or 3D autocorrelation reconstruction in combination with phase retrieval tomography.^17^ Also a diffuser in the pupil plane consisting of randomly placed microlenses with varying focal lengths has been implemented; in this case, the random positions provide a larger field of view compared to a conventional microlens array, and the diverse focal lengths improve the axial depth range.^18^ Another interesting approach is Fourier ptychographic microscopy, which iteratively stitches together a number of different angles illuminated, low-resolution intensity images in Fourier space to produce a wide-field, high-resolution complex sample image.^19^^,^^20^

On the other hand, multifocus (focus-stacking or z-stacking) microscopy^21^^,^^22^ is a simple technique where a scanning mechanism is introduced into a wide-field microscope in order to allow the acquisition of a set of differently focused images along the optical axis. Extended DoF (or all-in-focus) image is usually recovered using focus-recognition algorithms and depth-map retrieval.

As a way to overcome DoF limitation while achieving 3D visualization in fluorescence microscopy, in the present paper we propose a method based on multifocus sensing where a custom-built fluorescence microscope incorporating an electrically focus-tunable lens (EFTL) is employed to optically sweep in-depth the structure of MCTS. The EFTL allows a non-mechanical scanning in order to avoid lateral displacements between acquired images (neither the sample nor the optics are moved)^23^^,^^24^ and once multifocus images are taken, image registration is performed to match the different fields of view. Then a Fourier domain post-processing approach^25^^,^^26^—which does not require depth-map estimation or segmentation of in-focus regions—is applied and the acquired information is reorganized through algorithms to allow DoF extension, synthesis of novel viewpoints as well as reconstruction of stereoscopic pairs which can serve as 3D visualization tools of a thick biological sample. Validation experiments corresponding to 3D visualization of MCTS are presented.

## 2 Methodology

### 2.1 Multifocus Image Acquisition and Field-of-View Correction

**Fig. 1:**
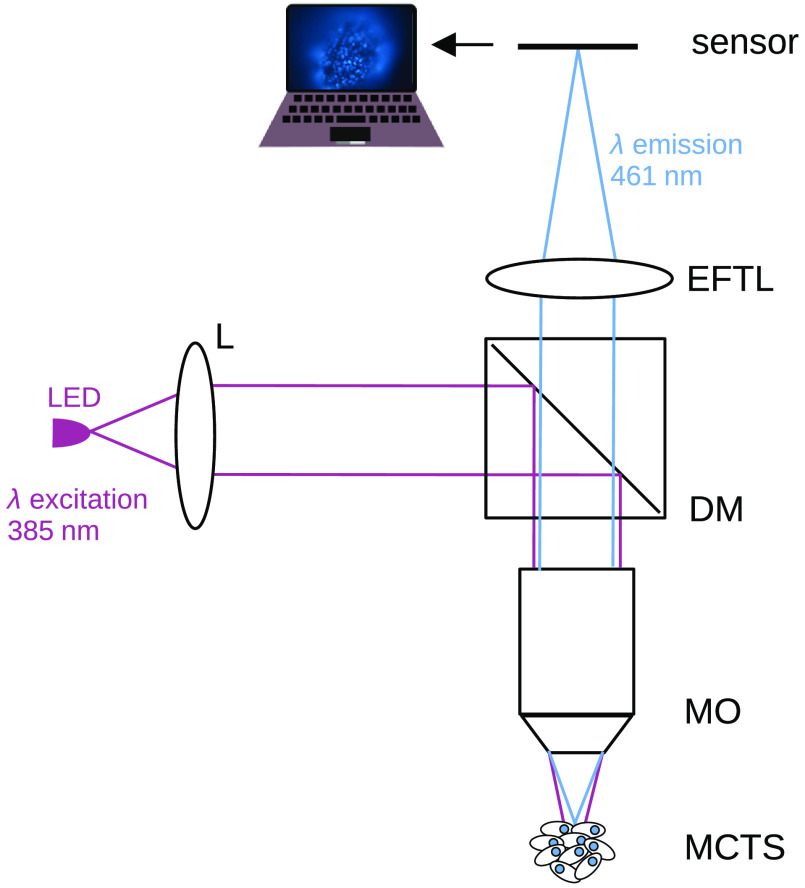
Scheme of the multifocus custom built fluorescence microscope. LED: Light emission diode as excitation source with center wavelength at 385 nm, L: illumination lens system, DM: dichroic mirror, MO: microscope objective, EFTL: electrically focus-tunable lens. MCTS: multicellular tumor spheroid stained with DAPI (emission peak centered at 461 nm).

The scheme of the custom-built fluorescence microscope is shown in Fig. 1. The main components include a camera sensor (Thorlabs CCD 8051-C USB 3.0, 3296×2472  pixels resolution, 5.5  μm pixel pitch), an EFTL (Optotune EL-16-40-TC-VIS-5D-C), a LED with an emission peak at 385 nm and FWHM of 12 nm (Thorlabs LED M385LP1), a dichroic mirror (reflection 375 to 393 nm, transmission 414 to 450 nm) and a water immersion microscope objective lens (Olympus UMPLFLN 20×, numerical aperture NA=0.5, focal length $f_{\mathrm{MO}}=9.00\;\mathrm{mm}$, working distance WD=3.5  mm).

The biological sample used in this work is from human prostatic carcinoma cell line (LNCaP)^27^ and was cultivated to form MCTS by means of the hanging drop method in which cells are suspended in droplets of medium where they develop into coherent 3D aggregates and are readily accessed for analysis.^28^ Cells were then stained with DAPI with a broadband excitation centered at 358 nm and emission at 461 nm. An extra filter centered at 457 nm and bandwidth 22 nm was placed before the sensor in order to enhance the contrast of the images.

Parts of the sample to be captured in-focus are those placed at the conjugate image plane of the sensor, which is shifted from the working distance plane of the microscope objective by an amount z given as

$$z=-\frac{f_{\mathrm{MO}}P(D-P^{-1})}{P^{-1}f_{\mathrm{MO}}^{-1}+(D-P^{-1})f_{\mathrm{eq}}^{-1}},$$ (1)

where $f_{\mathrm{eq}}$ is the equivalent focal length of the combination of the microscope objective and the EFTL and verifies

$$f_{\mathrm{eq}}^{-1}=f_{\mathrm{MO}}^{-1}+P-f_{\mathrm{MO}}^{-1}Pd,$$ (2)

while D is the distance between the EFTL and the sensor and d the distance between the back principal plane of the microscope objective and the EFTL. Optical power P of the EFTL can be varied between 3 and −2 diopters for currents between 270 and −230  mA. Since in our setup D≈10  cm, d≈5  cm, the maximum focusing (or z) range of the system is ∼210  μm.

In-focus parts of the sample are obtained at the sensor with lateral magnification M given as

$$M=P^{-1}f_{\mathrm{MO}}^{-1}+(D-P^{-1})f_{\mathrm{eq}}^{-1},$$ (3)

which varies along the focusing range with a maximum relative change of ∼15%. This change is reflected in turn in a change in the FoV along the images obtained while the current through the EFTL varies.

**Fig. 2:**
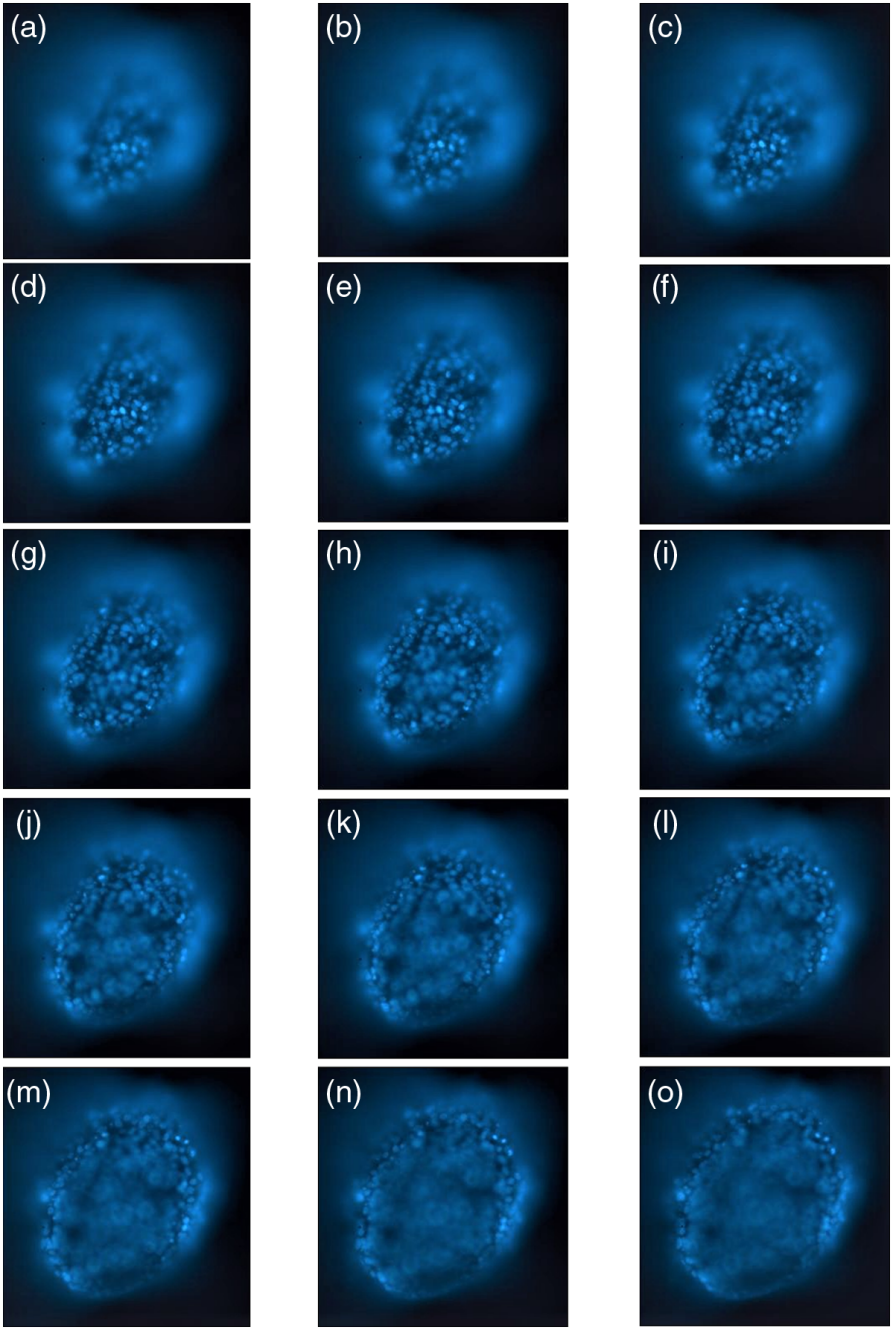
(a)–(o) 15 multifocus images acquired (z-stack) for currents in the EFTL between 265 and 125 mA in steps of −10  mA. See Video 1 for a visualization of the stack (Video 1, MP4, 0.2 MB [URL: https://doi.org/10.1117/1.JBO.27.6.066501.1]).

The multifocus stack was acquired for a set of currents in the EFTL $j_{k},k=1,\ldots ,N$ between 265 and 125 mA in steps of −10  mA. The N=15 image stack is shown in Fig. 2. Note that field of view is not constant along with the stack of images and needs to be corrected before the synthesis of novel viewpoints from multifocus stack is performed.^29^

Since in the present work the EFTL is positioned in the set-up in a way that total intensity remains constant between the acquired images (illumination path is not affected by the change in focus of the EFTL) this allows us to use conservation of energy (i.e., integral of intensity values in a given image of the stack should be constant) to implement registration between images of the stack (note that in other works including an EFTL,^24^ illumination intensity changes between the acquired images due to the position of the EFTL in the set-up, so conservation of radiant energy does not hold and registration needs to be performed following different approaches). Energy is evaluated for a given reference image (in our case k=1 image) and the rest of the images in the stack are rescaled to give the same value. Then the captured visual information is reorganized through a Fourier domain postprocessing approach which does not require depth-map estimation or segmentation of in-focus regions. Image reconstruction is accomplished considering only, besides an effective parameter, the current through the EFTL for each image in the stack.

### 2.2 Image Formation Model and Novel Viewpoint Synthesis

Once the multifocus image stack is acquired, postcapture processing algorithms enable the synthesis of images with novel viewpoints of the scene.^25^ Let $i_{k}$ be the intensity distribution of the k’th image of a stack of N images. [For color images in RGB space $i_{k}=(i_{k}^{R},i_{k}^{G},i_{k}^{B})$, k=1,…,N]. The $i_{k}$ image taken with current $j_{k}$ through the EFTL can be described, neglecting noise, and chromatic aberrations, by the following equation:

$$i_{k}(x,y)=f_{k}(x,y)+\underset{k^{\prime }\neq k}{\sum }h_{kk^{\prime }}(x,y)*f_{k^{\prime }}(x,y),$$ (4)

where $f_{k}$ is the in-focus region of $i_{k}$. The part of the scene that is out-of-focus in $i_{k}$ comes from the 2D convolution between $f_{k^{\prime }}$ (in-focus part of $i_{k^{\prime }}$) and the 2D intensity PSF $h_{kk^{\prime }}(x,y)$ associated with the currents $j_{k}$ and $j_{k^{\prime }}$

$$h_{kk^{\prime }}(x,y)=\frac{1}{\pi r_{kk^{\prime }}^{2}}\mathrm{circ}(\frac{\sqrt{x^{2}+y^{2}}}{r_{kk^{\prime }}}).$$ (5)

where

$$\frac{r_{kk^{\prime }}}{p}=R_{0}|j_{k}-j_{k^{\prime }}|,$$ (6)

and

$$R_{0}=\frac{R\alpha }{p},$$ (7)

where R is the aperture of the imaging system, α is the linear coefficient for the relation between lateral magnification and current through EFTL and p is the pixel pitch of the camera. For the stack of images in Fig. 2 effective parameter $R_{0}\approx 0.67\;{\mathrm{mA}}^{-1}$.

Let us consider the Fourier transform (FT) of the set of N coupled Eq. (4), and arrange them in vector form as

$$\overset{\to }{I}(u,v)=H(u,v)\overset{\to }{F}(u,v),$$ (8)

where (u,v) are spatial frequencies and N-element column vectors $\vec{I}$, $\vec{F}$ and N×N symmetric matrix H are given as

$$\overset{\to }{I}(u,v)=(\begin{array}{c} I_{1}(u,v)\\ I_{2}(u,v)\\ \vdots \\ I_{N}(u,v) \end{array}),\overset{\to }{F}(u,v)=(\begin{array}{c} F_{1}(u,v)\\ F_{2}(u,v)\\ \vdots \\ F_{N}(u,v) \end{array}),\;H(u,v)=(\begin{array}{cccc} 1 & H_{12}(u,v) & \ldots & H_{1\;N}(u,v)\\ H_{12}(u,v) & 1 & & \vdots \\ \vdots & & \ddots & H_{N-1\;N}(u,v)\\ H_{1\;N}(u,v) & \ldots & H_{N-1\;N}(u,v) & 1 \end{array}).$$ (9)

If H(u,v) is invertible, then the solution to the linear system given by Eq. (8) is $\vec{F}(u,v)=H^{-1}(u,v)\vec{I}(u,v)$, but if H(u,v) is not invertible (as for the DC frequency components), then a solution to the system may be found through the Moore–Penrose pseudoinverse $H^{†}$.^30^ The Moore–Penrose pseudoinverse provides the set of vectors that minimize the Euclidean norm $\left\|H(u,v)\vec{F}(u,v)-\vec{I}(u,v)\right\|$ in the least squares sense. Thus, the minimal norm vector is given as

$$\overset{\to }{F}(u,v)=H^{†}(u,v)\overset{\to }{I}(u,v).$$ (10)

The reconstruction of an arbitrary horizontal viewpoint of the scene is accomplished by simulating the displacement $b_{x}$ of a pinhole camera in the horizontal direction with respect to the center of the original pupil (similarly for a $b_{y}$ displacement in the vertical direction). The horizontal disparity $d_{k}$ between the images of a given point of the in-focus component $f_{k}$ as seen by the sensor of a centered pinhole camera and a pinhole camera displaced to the left is given as

$$d_{k}=b_{x}\alpha j_{k},$$ (11)

(aside from a constant factor independent of k and related to the magnification at zero current). Then, in the piecewise planar approximation of the 3D scene, to obtain a shifted viewpoint $s_{b_{x}}(x,y)$, each focus slice $f_{k}(x,y)$ should be shifted in an amount according to the disparity associated with the $j_{k}$ current through EFTL and the baseline displacement $\left(b_{x}\right)$ of the camera

$$s_{b_{x}}(x,y)=\overset{N}{\underset{k=1}{\sum }}f_{k}(x-b_{x}\alpha j_{k},y).$$ (12)

In particular, $s_{0}(x,y)$ recovers the image as captured with a pinhole camera in the center of the original circular pupil (i.e., extended-DoF or all-in-focus image reconstruction of the scene^23^).

By means of the FT shift theorem, which states that translation in the space domain introduces a linear phase shift in the frequency domain,^31^ and by using Eq. (10) for $\vec{F}(u,v)$, we obtain the FT of Eq. (12):

$$S_{b_{x}}(u,v)=\overset{N}{\underset{k=1}{\sum }}e^{-j2\pi \alpha j_{k}(b_{x}u)}{\left(H^{†}(u,v)\overset{\to }{I}(u,v)\right)}_{k}.$$ (13)

Let us now consider the baseline displacement $b_{x}$ as a fraction $\beta_{x}$ ($|\beta_{x}|\leq 1$) of the pupil R (since displacements outside of the aperture have no physical meaning)

$$b_{x}=\beta_{x}R,$$ (14)

so by means of Eq. (7), Eq. (13) can be rewritten as

$$S_{\beta_{x}}(u,v)=\overset{N}{\underset{k=1}{\sum }}e^{-j2\pi j_{k}\beta_{x}R_{0}(pu)}{\left(H^{†}(u,v)\overset{\to }{I}(u,v)\right)}_{k}.$$ (15)

Finally, by Fourier inverse transform of Eq. (15) we obtain the new scene perspective as seen from a pinhole camera, translated a fraction $\beta_{x}$ to the left of the center of the original circular pupil^25^

$$s_{\beta_{x}}=F^{-1}\{S_{\beta_{x}}\}.$$ (16)

In order to achieve visualization with full parallax, it is straightforward to extend Eq. (15) to the case of vertical motion simulation

$$S_{\beta_{y}}(u,v)=\overset{N}{\underset{k=1}{\sum }}e^{-j2\pi j_{k}\beta_{y}R_{0}(pv)}{\left(H^{†}(u,v)\overset{\to }{I}(u,v)\right)}_{k},$$ (17)

and consider the synthesis of new scene perspective as seen from a pinhole camera, translated a fraction $\beta_{y}$ upward of the center of the original circular pupil by Fourier inverse transform of Eq. (17)

$$s_{\beta_{y}}=F^{-1}\{S_{\beta_{y}}\}.$$ (18)

The proposed method is then able to reconstruct the extended DoF and allows the visualization of the reconstructed scene from different perspectives without previous segmentation of the focused regions from the images in the stack. However, it is possible to retrieve the depth map by combining this method with other schemes.^32^

## 3 Results

### 3.1 Extend Depth-of-Field and Viewpoint Synthesis

**Fig. 3:**
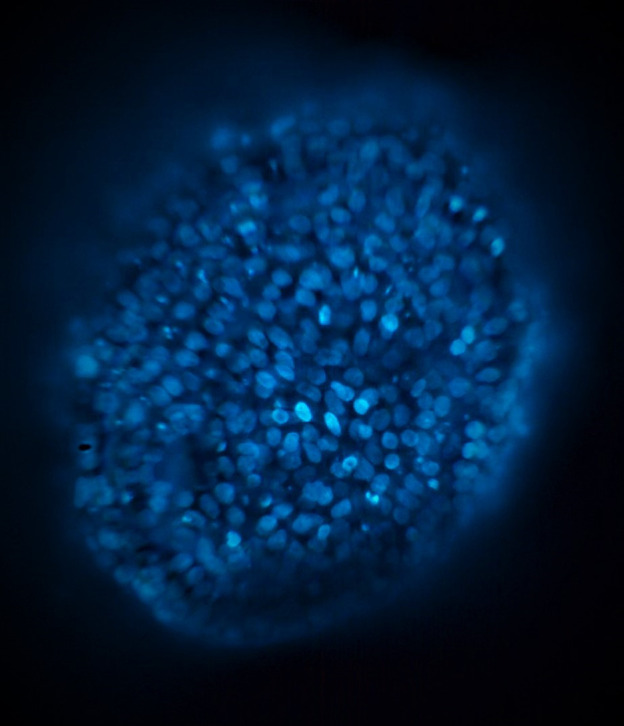
Extended DoF for a virtual centered pinhole view ($\beta_{x}=0$, $\beta_{y}=0$); see Video 2 for a complete set of novel viewpoints corresponding to $-0.25\leq \beta_{x}\leq 0.25$ and $-0.25\leq \beta_{y}\leq 0.25$ (Video 2, MP4, 1.0 MB [URL: https://doi.org/10.1117/1.JBO.27.6.066501.2]).).

Reconstruction of the extended DoF or all-in-focus image corresponds to $\beta_{x}=0$, (i.e., as seen with a centered pinhole camera) and is shown in Fig. 3. Note how, unlike original images of the stack in Fig. 2, individual cell nuclei from different depths of the aggregate can be clearly seen in a single image.

If we instead consider arbitrary fractional displacements $\beta_{x}$, $\beta_{y}$, the corresponding viewpoints can be synthesized from Eqs. (16) and (18), respectively (combination of horizontal and vertical viewpoints is straightforward). A complete set of novel viewpoints for $-0.25\leq \beta_{x}\leq 0.25$, $-0.25\leq \beta_{y}\leq 0.25$ is available in Video 2.

### 3.2 Stereoscopic Pairs for 3D Visualization

Binocular vision is based on the fact that 3D objects are perceived from two different perspectives due to the horizontal separation between our left and right eyes. As a result, the left and right images of a 3D scene in our retinas are slightly different. This retinal disparity between the images provides the observer with information about the relative distances and depth structure of 3D objects. Both perspectives of the same 3D scene are fused by the brain to give the perception of depth.^33^^,^^34^

In a similar way, a pair of stereoscopic images can be generated by considering a virtual stereocamera^35^ formed by a left pinhole camera displaced to the left of the center of the original pupil, $b_{x}=B/2$, and a right pinhole camera displaced to the right of the center of the original pupil, $b_{x}=-B/2$, where the separation B between the left and right virtual pinhole cameras is known as the baseline. Since points of view from outside of the aperture have no physical meaning, B≤2R.

**Fig. 4:**
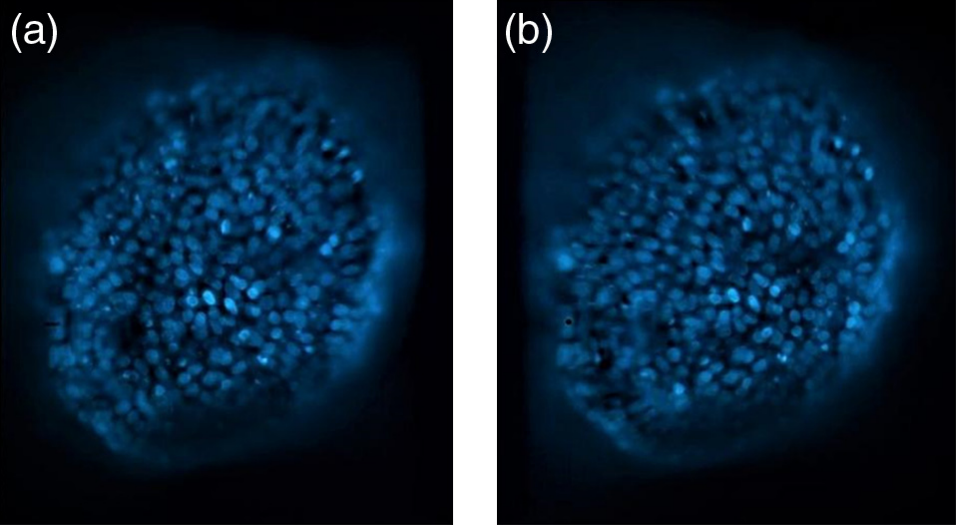
Stereoscopic pair for cross eyed visualization. (a) Reconstructed and (b) perspective images [images corresponding to pinhole virtually displaced to the right and to the left, respectively].

Then, it is straightforward to reconstruct the left and right views according to

$$i_{L}(x,y)=s_{B/2R}(x,y),$$ (19)

$$i_{R}(x,y)=s_{-B/2R}(x,y),$$ (20)

where each r.h.s. is to be calculated by means of Eq. (16). Once the stereoscopic pair is generated, the left and right images can be displayed in different ways.^36^ In Fig. 4, the cross-eye stereo pair for B=R/2 is presented. With some practice, the fused image is perceived by deliberately crossing one’s eyes until the two images come together.

### 3.3 Performance Assessment

**Fig. 5:**
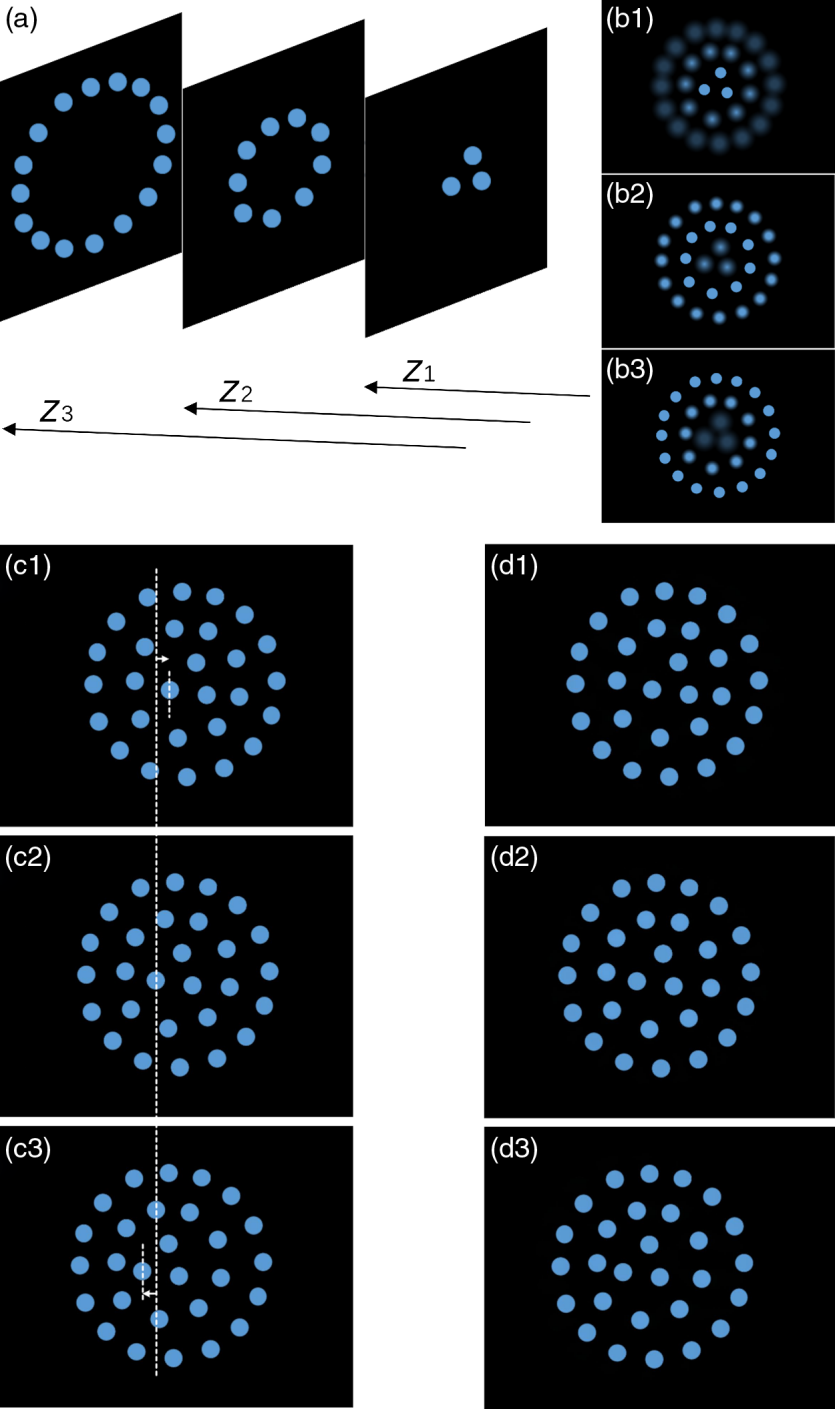
Synthetic stack of fluorescent beads and reconstruction of viewpoints. (a) 3D scene. ($b_{1-3}$) Images of the stack corresponding to $R_{0}\approx 12.7\;{\mathrm{mA}}^{-1}$ and the system focusing for currents through the EFTL 50, 33.3, 0 mA, respectively. ($c_{1-3}$) Ground-truth for fractional displacements $\beta_{x}=-0.5,0,0.5$, respectively [vertical dashed guideline passing through a bead in the central viewpoint ($b_{2}$) added to visualize more clearly the displacements]. ($d_{1-3}$) Reconstructed viewpoints for fractional displacements $\beta_{x}=-0.5,0,0.5$, respectively.

Quantitative comparison can be performed with the help of a synthetic multifocus stack since, unlike the real stack, a ground-truth reference for each point of view of interest can be constructed. Figure 5(a) shows the synthetic 3D scene representing three rings of fluorescent beads with each ring lying on a different plane at distances $z_{i},i=1,2,3$. Images of the stack corresponding to the system focusing on each of these planes (for currents $j_{i},i=1,2,3$) are shown in Fig. 5($b_{1-3}$), respectively.

Figure 5($c_{1-3}$) shows the ground truth for the scene as viewed from a pinhole camera displaced to the left, center, and right, for relative displacements $\beta_{x}=0.5,0,-0.5$, respectively. The multifocus stack of Fig. 5($b_{1-3}$) is used to render the same viewpoints and the results obtained by means of Eq. (16) are presented in Fig. 5($d_{1-3}$), respectively. Table 1 shows the mean square error resulting from the comparison (of luminances) against the ground truth for each relative displacement, showing a very good agreement between the reconstructed viewpoint and its corresponding ground truth.

**Table 1:** MSE values from comparison against ground-truth for different horizontal relative displacements $\beta_{x}$.

$\beta_{x}$	MSE
+0.5	0.4865
0	0.7065
−0.5	0.4799

## 4 Conclusion

We have developed a custom-built fluorescence microscope that incorporates an electrically focus-tunable lens and allows us to acquire sets of multifocus images from thick biological samples, in particular MCTS.

Our algorithms operated then over the acquired stacks to accomplish extended DoF by multifocus image fusion without depth-map estimation or segmentation of the in-focus regions.

Besides all in focus reconstruction along the optical axis, viewpoint synthesis with shifts in perspective can be performed to provide a stereoscopic pair of images of the sample as well as 3D visualization of the 3D structure of the cell aggregates.

Our proof-of-principle experimental results show the potential of the present approach, which could serve in a wide range of biological and biomedical applications where 3D visualization of a biological sample might be useful. As a future line of work, it might be interesting to include more fluorescent channels to assess different cellular structures.
